# Co-expression of Interleukin-15 Enhances the Protective Immune Responses Induced by Immunization with a Murine Malaria MVA-Based Vaccine Encoding the Circumsporozoite Protein

**DOI:** 10.1371/journal.pone.0141141

**Published:** 2015-10-27

**Authors:** Marcela Parra, Xia Liu, Steven C. Derrick, Amy Yang, Alvaro Molina-Cruz, Carolina Barillas-Mury, Hong Zheng, Phuong Thao Pham, Martha Sedegah, Arnel Belmonte, Dianne D. Litilit, Thomas A. Waldmann, Sanjai Kumar, Sheldon L. Morris, Liyanage P. Perera

**Affiliations:** 1 Food and Drug Administration, Center for Biologics Evaluation and Research, Silver Spring, MD, 20993, United States of America; 2 Laboratory of Malaria and Vector Research, National Institute of Allergy and Infectious Diseases, Rockville, MD, 20852, United States of America; 3 Naval Medical Research Center, Silver Spring, MD, 20910, United States of America; 4 National Cancer Institute, Bethesda, MD, 20892, United States of America; Agency for Science, Technology and Research - Singapore Immunology Network, SINGAPORE

## Abstract

Malaria remains a major global public health problem with an estimated 200 million cases detected in 2012. Although the most advanced candidate malaria vaccine (RTS,S) has shown promise in clinical trials, its modest efficacy and durability have created uncertainty about the impact of RTS,S immunization (when used alone) on global malaria transmission. Here we describe the development and characterization of a novel modified vaccinia virus Ankara (MVA)–based malaria vaccine which co-expresses the *Plasmodium yoelii* circumsporozoite protein (CSP) and IL-15. Vaccination/challenge studies showed that C57BL/6 mice immunized with the MVA-CSP/IL15 vaccine were protected significantly better against a *P*. *yoelii* 17XNL sporozoite challenge than either mice immunized with an MVA vaccine expressing only CSP or naïve controls. Importantly, the levels of total anti-CSP IgG were elevated about 100-fold for the MVA-CSP/IL15 immunized group compared to mice immunized with the MVA-CSP construct that does not express IL-15. Among the IgG subtypes, the IL-15 expressing MVA-CSP vaccine induced levels of IgG1 (8 fold) and IgG2b (80 fold) higher than the MVA-CSP construct. The significantly enhanced humoral responses and protection detected after immunization with the MVA-CSP/IL15 vaccine suggest that this IL-15 expressing MVA construct could be considered in the development of future malaria immunization strategies.

## Introduction

Despite improved vector control strategies and relatively effective antimalarial drug regimens, malaria remains one of world’s most deadly pathogens. In 2012, malaria infections were responsible for an estimated 627,000 deaths; most of this mortality was seen in young African children [1]. Although malaria treatment and vector control efforts have lowered the overall toll of this disease, the long-term and most cost-effective solution for controlling the malaria epidemic is expected to be by prevention through the development of a highly efficacious vaccine. However, the development of effective malaria vaccines has been extremely challenging [2]. In endemic areas, despite repeated infections with this parasite, sterilizing immunity is not generated and even persons with clinical immunity can carry a low-grade parasite burden and hence serve as reservoirs for further transmission. Furthermore, the malaria life cycle is complex and the protective immune responses required to inhibit parasite proliferation have not been clearly defined [3, 4].

The most advanced malaria vaccine candidate is RTS,S, a pre-erythrocytic vaccine consisting of a *P*. *falciparum* circumsporozoite protein (CSP) fused to the hepatitis B S-protein formulated in AS01 adjuvant [5]. Thus far, the clinical studies using the RTS,S formulation have yielded mixed results. Recent trials have shown the RTS,S vaccine is relatively safe and has moderate efficacy against clinical malaria (55.8% in 5–17 months old children) at 12 months after a third immunization [6]. However, in the same age group, the vaccine-induced protective responses declined significantly at 48 months post-vaccination to 28.3%. Importantly, vaccine efficacy increased in these young children who were boosted at 20 months with RTS,S to 36.3% at the 48 month time point. The effectiveness of immunization with three doses of the RTS,S vaccine has been shown to be even lower in infants that are 6–12 weeks of age (18.3% at 38 months). In these young infants, boosting at 20 months only increased the vaccine efficacy to 25.9% [7]. Interestingly, analysis of data from the clinical trials has suggested that RTS,S vaccine-induced protection correlates with anti-CSP antibody and CD4 T cell responses [8]. While the initial clinical study results are encouraging, the RTS,S immunization regimen clearly needs to be optimized and the success of future CSP-based vaccination approaches will likely be dependent on amplifying the strength and durability of antibody and CD4 T cell responses against CSP.

Since clinical immunization approaches have generally been insufficient in the development of vaccines against intracellular pathogens, prime/boost vaccination regimens are being developed and tested. By combining partially effective vaccines, prime/boost approaches can evoke superior levels of protective immunity compared to individual vaccination regimens [9]. Among the most powerful immunization boosting agents are recombinant MVA vaccines. In addition to being extremely safe, MVA vaccines have consistently been shown to boost primed T and B cell responses against intracellular pathogens such as HIV and *Mycobacterium tuberculosis* [10–15]. In animals and humans, a recombinant MVA vaccine expressing the malaria CSP antigen was well tolerated and induced significant cellular and humoral immune responses [15, 16]. Disappointingly, in a prime/boost study of 18 adults, boosting a priming RTS,S vaccination with the MVA-CSP construct did not lead to enhanced protection against a controlled human malaria challenge [17].

Recently, recombinant MVA constructs that co-express IL-15 as well as *M*. *tuberculosis*, HIV, or influenza antigens have been generated and characterized [10,–12, 18]. IL-15 has an important adjuvant-like impact on innate and adaptive immunity because of its modulatory effects on immune cells [19]. In addition to promoting T cell and natural killer cell proliferation, IL-15 can play a role in the maintenance of established memory cell populations and the differentiation of B cells. Co-expression of IL-15 in MVA-based constructs has generally resulted in strong and persistent humoral and cellular responses against transgene antigens [10, 11]. In this study, we created an MVA-based vaccine that co-expresses IL-15 and the *Plasmodium yoelii* CSP. Here we show that this novel MVA-CSP/IL-15 construct induces stronger humoral responses and significantly better protective immunity than a recombinant MVA vaccine that expresses only *P*. *yoelii* CSP.

## Materials and Methods

### Animals

C57BL/6 mice of 6–8 weeks of age were purchased from the Jackson Laboratories (Bar Harbor, ME). This study was done under the guidelines for the care and use of laboratory animals specified by the National Institutes of Health. The experimental procedures were approved by the Institutional Animal Care and Use Committee of the Center for Biologics Evaluation and Research under Animal Study Protocol 2008–08.

#### MVA-CSP vaccines

The full length *P*. *yoelii* CSP gene was integrated into the HA locus of modified vaccinia virus Ankara (MVA) virus such that the CSP gene expression is driven from a synthetic early-late hybrid vaccinia promoter and the recombinant virus was isolated under mycophenolic acid selection. The MVA-CSP/IL15 vaccine was generated by cloning the Py NL CSP gene as well as the human interleukin-15 (IL-15) gene into MVA vector. The MVA-CSP vaccine was created similarly as the MVA-CSP/IL-15 but without the IL-15 gene. The expression of *P*. *yoelii* CSP was confirmed by Western blot using a polyclonal anti *P*. *yoelii* CSP mouse antibody after infection of BHK21 cells. IL-15 expression in BHK21 cells infected with the MVA vaccines was evaluated using an IL-15 ELISA as described previously [18].

#### Parasites

*P*. *yoelii* sporozoites (17 XNL, nonlethal strain, clone 1.1) were generated through transmission of infection in *Anopheles stephensi* mosquitoes. Briefly, mosquitoes were fed on CD1 mice infected with blood stage *P*. *yoelii* 17XNL parasites and *P*. *yoelii* sporozoites were obtained by dissection of mosquito salivary glands isolated on day 14 post-infectious blood meal in M199 medium containing 5% mouse serum. For challenge studies, sporozoites were counted using a hemocytometer and diluted to a final concentration of 100 parasites per 200 μl in M199 medium.

#### Immunzations and evaluation of vaccine efficacy

Five to 10 mice per group were vaccinated subcutaneously with either the MVA-CSP/IL15 vaccine (1x10^7^pfu/100ul) or the MVA-CSP construct (1x10^7^pfu/100μl). In each experiment, five to ten non-vaccinated mice were used as controls. Following an intravenous (i.v) challenge with 100 *P*. *yoelii* sporozoites, vaccine efficacy was measured by measuring parasite burden by microscopy on Giemsa-stained blood films beginning from day 7 post-challenge infection and then continued every three days till blood stage infections were cleared. A minimum of 20 independent fields were examined per smear and approximately 1000 RBCs were counted per smear. For an experiment to be considered valid, the blood stage infection induced by the i.v injection of 100 *P*. *yoelii* sporozoites had to reach parasitemia levels equal to or greater than 5% of infected red blood cells in the control mice.

#### Cell depletion studies

To identify the cell subsets that mediated the vaccine-induced protective responses, mice were treated 4 and 1 day before a *P*. *yoelii* sporozoite challenge with either anti-CD4 (clone GK1.5, rat anti-mouse IgG2b), anti-CD8 (clone 2.43, rat anti-mouse IgG2b), or anti-NK1.1 (clone PK.136, anti-mouse IgG2a,k) antibodies. The parasitemia levels were monitored as described above.

#### Liver stage P. yoelii 17XNL parasite growth inhibition assay

For the evaluation of MVA-CSP vaccine on the growth of PyNL parasites inside the liver cells, C57BL/6 mice were vaccinated with MVA-CSP/IL15 (1x10^7^pfu in 100μl per mouse) and control mice were sham vaccinated with PBS. Four weeks after the vaccination, mice were injected with 6,000 sporozoites of *P*. *yoelii* 17XNL strain expressing the Green Fluorescence Protein (GFP) designated as GFP-PyNL sporozoite (a kind gift from Ana Rodriguez, NYU) in a volume of 100 microliters intravenously. GFP-PyNL sporozoites were generated in *A*. *stephensi* mosquitoes and purified by dissection of salivary glands. Livers were harvested 24 hours post injection and immediately subjected to 2-photon microscopy.

#### Flow cytometry assessment of vaccine-induced T cells

Four vaccinated and control mice were used to determine the frequencies of CD4^+^ and CD8^+^ T cells expressing IFNγ, TNF-α, and/or IL-2 at one month post-vaccination. Spleen cells were isolated and the resulting single cell suspension was centrifuged to pellet the cells and treated with ACK lysing buffer for 5 minutes. After washing, the cells were plated in a 24 well plate at 5x10^6^ spleen cells per/0.5 ml/well. *P*. *yoelli* NL rCSP at 2μg/0.5ml was then added to corresponding wells for a final volume of 1 ml per well. After incubating the plates for 48 hours, the cells were treated for 4–5 hours with Golgi plug (BD Biosciences). The cells were then harvested, washed, pelleted, counted and prepared for flow cytometry. Next, the cells were transferred to 12 x 75 mm tubes, washed with PBS and resuspended in ~50 μl PBS. Live-Dead stain (Invitrogen, Carlsbad, CA) (10 μl of a 1:100 dilution) was added to each tube and incubated for 30 min. at room temperature to allow gating on viable cells. After washing the cells with PBS-2% FBS, antibody against surface CD16/CD32 (FcγIII/II receptor, clone 2.4G2) (Fc block) was added in a volume of ~50 μl and incubated at 4°C for 15 min. The cells were then stained for 30 min. at 4°C by adding antibodies against the CD4 (rat anti-mouse CD4 Alexa Fluor 700 [AF-700] Ab, clone RM4-5) protein at 0.1 μg per tube. Cells were also stained against the CD8 (rat anti-mouse CD8 peridinin chlorophyll protein complex [PerCP] Ab, clone 53–6.7) protein at 0.4μg per tube. Following incubation, the cells were washed twice with PBS and then fixed for 30 min. at 4°C with 2% paraformaldehyde in PBS. After fixing, the cells were pelleted and washed twice with PBS-FBS. Fixed cells were washed twice with perm-wash buffer (1% FBS, 0.01 M HEPES, 0.1% saponin in PBS) followed by intracellular staining using the following antibodies at 0.2 μg per tube: rat anti-mouse IFN-γ phycoerythrin [PE] Ab, clone XMG1.2; rat anti-mouse TNF-α fluorescein isothiocyanate [FITC] Ab, clone MP6-XT22; rat anti-mouse IL-2 allophycocyanin [APC] Ab, clone JES6-5H4. The cells were incubated at 4°C for 30 min., washed twice with perm-wash buffer and then twice with PBS-FBS. All antibodies were obtained from BD Biosciences.

The frequency of monofunctional IFN-γ producing T cells and the different multifunctional T cell subsets were determined using a LSRII flow cytometer (Becton Dickinson) and FlowJo software (Tree Star Inc., Ashland, Oregon). We acquired 250,000 events per sample and then, using FlowJo, gated on live, single cell lymphocytes. To determine the frequency of different populations of multi-functional T cells (MFT cells), we gated on CD4 or CD8 T cells staining positive for TNF-α and IFN-γ, TNF-α and IL-2, IFN-γ and IL-2 or all three cytokines.

#### Evaluation of anti-CSP antibody levels

Serum samples were collected two weeks post-vaccination for the antibody response measurements. Antibody levels were evaluated using an anti-CSP ELISA procedure. The ELISA plate was coated with recombinant *P*. *yoelii* CSP protein (CSP at a concentration of 8 μg/ml dissolved in a coating buffer containing 15 mM sodium carbonate and 35 mM sodium bicarbonate, pH 9.6; 100 μl/well) and incubated overnight at 4°C. After washing three times with wash buffer (PBS/Tween-20), blocking buffer (5% milk in PBS) was added for one hour and then 100μl of individual serum at a ratio 1:25 to 1:12800 was added for two hours at room temperature. Following additional washes, the plates were incubated with the respective second antibodies (IgG, IgG1, IgG2b, IgG2c, IgG3 or IgM, from Southern Biotechnology Associates) at a ratio of 1:2000 for 1 hour at room temperature. After washing again three times, 100μl ABTS substrate was added, incubated for 30 min, and plate was read at wavelength 405/650 nm on a VERSA max microplate reader. The endpoint titer was defined as the experimental mean + 3 standard deviations absorbance value that did not exceed the mean naïve absorbance value.

#### Statistical analyses

The protection and flow cytometry data were evaluated using Mann-Whitney test (for parametric data without normality datasets) or alternatively, Kruskal–Wallis 1-way analysis of variance (ANOVA) for multiple comparisons of nonparametric data followed by post hoc analysis using Tukey’s multiple comparison test as appropriate using Prism 5 program (GraphPad) and values of *P*<0.05 were considered statistically significant.

## Results

### Co-expression of *P*. *yoelii* CSP and IL-15 from the MVA-CSP/IL15 vaccine

**Fig 1:**
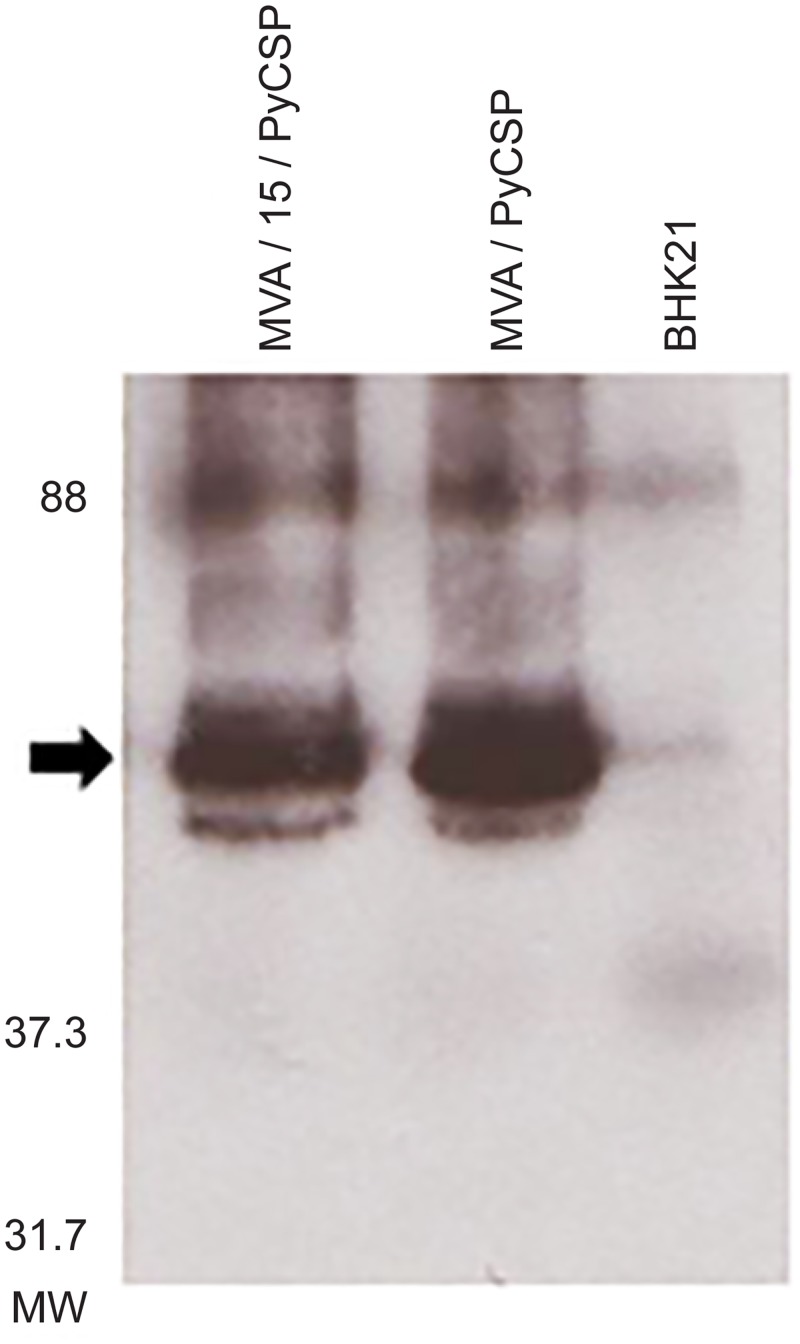
Expression of *P*. *yoelii* CSP in BHK 21 cells infected with MVA-CSP/IL15 and MVA-CSP vaccines. BHK 21 cells were infected with the respective MVA vaccines at a MOI of 10 and the infected cell lysates (150 μg) were evaluated for CSP expression seventy two hours later by western blotting using an anti-CSP antibody. The first two lanes were loaded with the MVA-CSP/IL-15 and MVA-CSP infected cell lysates respectively and the third lane was loaded with an equal amount of cell lysate (150 μg) prepared from uninfected BHK21. The arrowhead indicates the position of *P*. *yoelii* CSP and protein molecular weight markers are shown on the left.

The co-expression of *the P*. *yoelii* CSP and IL-15 from the MVA-CSP/IL-15 and MVA-CSP viral vectored vaccines was evaluated in BHK21 cell cultures. Seventy two hours after infection with the viral constructs, BHK 21 cells were lysed and the expression of CSP was assessed by western blot with a polyclonal anti-CSP serum. As seen in Fig 1, similar amounts of CSP were detected in lysates from cells infected with both MVA-CSP vaccines while CSP expression was not seen in uninfected BHK 21 cells. To quantitate IL-15 expression from the MVA constructs, BHK21 cell supernatants were analyzed using a human IL-15 ELISA kit. This analysis showed considerable IL-15 expression from the MVA-CSP/IL-15 vaccine. When BHK21 cells were infected at a MOI (multiplicity of infection) of 10, the culture supernatant of MVA-CSP/IL-15 infected cells contained approximately 2ng/ml of human IL-15 whereas the culture supernatant of MVA-CSP infected cells had no detectable human IL-15 (data not shown).

### Immunization with the MVA-CSP/IL15 vaccine protects against *P*. *yoelii* sporozoite challenge

**Fig 2:**
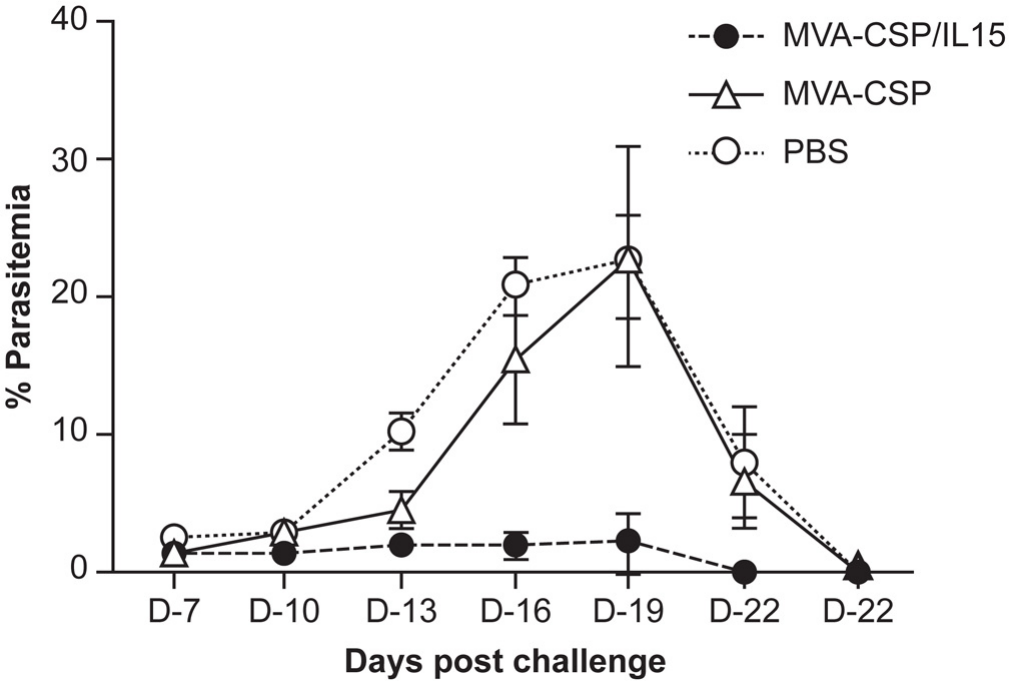
Representative parasitemia curves after infection of MVA-CSP/IL15, MVA-CSP vaccinated and naïve mice with 100 PyNL SPZ. MVA-CSP/IL15 vaccinated (closed circles), MVA-CSP vaccinated (open triangle) and naïve/PBS control (open circles) mice were infected intravenously with PyNL SPZ and parasitemias were evaluated by blood smears every third day starting at day 7. Results are expressed as mean % parasitemia ± SEM for ten mice per group. Differences in parasitemia levels among groups were significant at D-16 with a *p = 0.005, D-19 with a **p = 0.010 and D-22 with a ***p = 0.009 (Kruskal-Wallis test).

To assess whether the MVA-CSP/IL15 construct induces anti-sporozoite immunity and if the IL-15 expression enhanced protection, mice were immunized twice (one month apart) with the MVA-CSP/IL15 and MVA-CSP vaccines and then challenged one month later with PyNL sporozoites. Malaria parasitemia levels were evaluated in the blood smears every third day starting at day 7. Representative parasitemia curves for the three experiments in which peak parasitemias exceeded 5% are presented in Fig 2. The results for the non-vaccinated mice showed a typical *P*. *yoelii* parasitemia profile with peak values seen at approximately 16–19 days post-infection and parasite clearance by day 25. Interestingly, no significant differences in the parasitemia values (relative to naïve controls) were detected in mice that had been vaccinated with the MVA-CSP vaccine. In contrast, much lower parasitemia values were seen in mice vaccinated with the MVA-CSP/15 construct. Although this vaccine does not induce sterile immunity, immunization with the MVA-CSP/IL15 vaccine had an impact on the blood stage parasite burden relative to MVA-CSP and naïve controls. While no statistical differences in parasitemia levels were observed early in the ascending phase, more than 90% reduction in malaria parasitemia was observed at days 16 and 19 after the challenge in mice vaccinated with the MVA-CSP/IL15 construct compared to the other groups. For example, the parasitemia values at day 16 were 20.09 ± 2.1% for non-immunized and 15.5± 4.7% for MVA-CSP vaccinated mice versus 1.9± 0.9% for the MVA-CSP/IL15 group (p = 0.0057). In addition, compared to MVA-CSP and naïve control groups accelerating parasite clearance was observed for MVA-CSP/IL15 vaccinated mice. At day 22, parasitemia levels of approximately 8.0% were seen for the naïve controls (8.07± 3.9%) and the MVA-CSP group (6.7± 4.3%) while the parasitemia was essentially cleared (0.01± 0.01%) for the MVA-CSP/IL15 vaccinated mice.

### MVA-CSP/IL15 vaccine inhibits the growth of GFP-PyNL liver stage parasites

**Fig 3:**
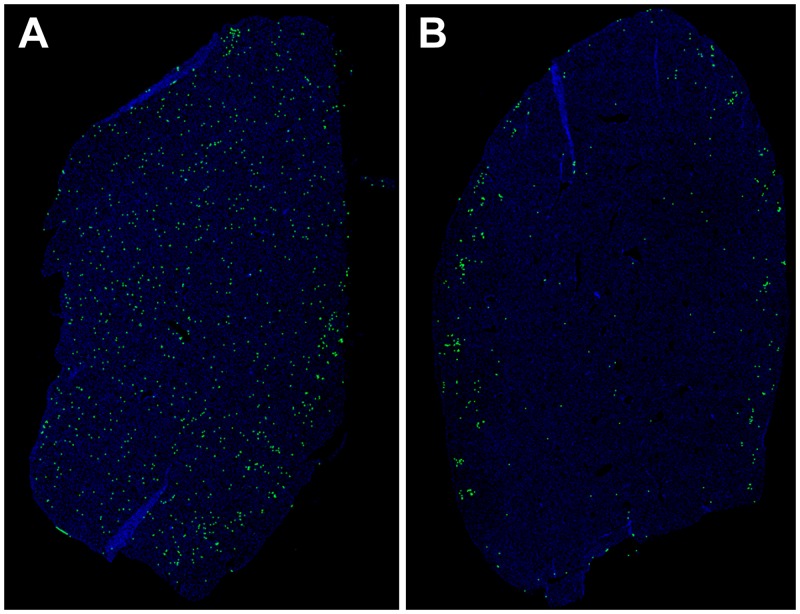
Liver sections from mice infected with GFP-expressing *P*. *yoelii* SPZ. Naïve controls (A) and mice immunized with a single dose of MVA-CSP/IL15 vaccine (B) were infected intravenously with GFP-expressing Py SPZ 4 weeks after the vaccination (6000 SPZ/mouse). Livers were harvested 24 hours post-infection and subjected to 2-photon microscopy. Fluorescent green spots represent hepatocytes harboring GFP-expressing *P*. *yoelii*.

To further examine whether MVA-CSP/IL15 vaccine reduced the blood stage parasite burden, the impact of vaccination on the growth of the liver stage GFP-PyNL parasites was evaluated by examining liver sections from MVA-CSP/IL15 vaccinated and control mice (2 mice per group) after a GFP- PyNL sporozoite challenge by intravenous route. Liver sections were prepared 24 hours after the infection and the sections were subsequently scanned microscopically using a 20X objective. As seen in the Fig 3, the numbers of GFP-PyNL sporozoites detected in the livers of naïve mice greatly exceeded the numbers observed in liver sections from vaccinated mice. In these representative sections, visual counting of fluorescent spots that represent individual liver stage GFP-PyNL parasites resulted in 765 spots in the naïve liver sections and 204 spots in the vaccinated sections, thus showing a 74% reduction in the liver stage parasite burden in the vaccinated sections. These results clearly demonstrated that MV-CSP/IL15 vaccine is highly effective in killing the liver stage parasite after a challenge infection with PyNL sporozoites.

### CD4 T cells contribute to the protective immunity induced by the MVA-CSP/IL15 vaccine

**Fig 4:**
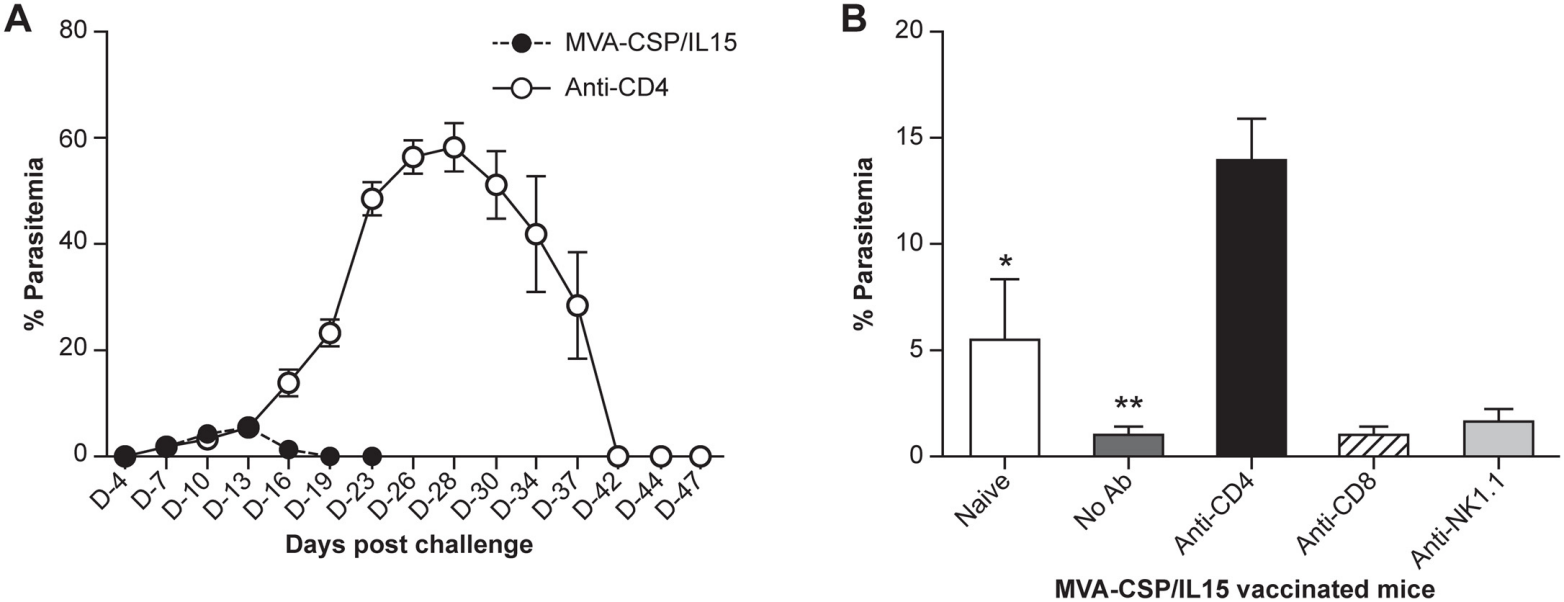
Parasitemia levels in infected, vaccinated mice depleted of immune cells. Panel **A**. Mice immunized with the MVA-CSP/IL15 vaccine were depleted of CD4 T cells (closed circles) using specific antibodies and then infected with PyNL SPZ before evaluating parasitemia levels. Control mice were vaccinated but were not depleted of CD4 T cells before the PyNL infection (closed circles). Results are expressed as the mean % parasitemia ± SEM (10 mice per group); p = 0.008 (Mann Whitney test). Panel **B**. Parasitemia levels at day 16 after the PyNL SPZ infection. Mice (n = 10) were immunized with the MVA-CSP/IL15 vaccine and then were treated with specific antibodies to deplete CD4 T cells, CD8 T cells or NK cells. The control groups consisted of non-depleted, vaccinated mice or naïve animals. At day 16 when parasitemia levels for all groups were compared (Kruskal-Wallis test), a statistical significance of p<0.05 was observed. Parasitemia levels for the CD4 T cell depleted vaccinated mice were significantly higher than the naïve levels (**p = 0.007) while the difference in the extent of parasitemia approached statistical significance [*p = 0.047 for the vaccinated compared to naïve mice (Mann Whitney test)].

To identify the immune cell subsets contributing to vaccine-induced protection, cellular depletion experiments were conducted. First, mice (10 per group) immunized with the MVA-CSP/IL15 vaccine were treated 1 month post-vaccination with an antibody that depleted greater than 95% of CD4 T cells. As seen in Fig 4A, vaccinated mice lacking CD4 T cells struggled to control the PyNL sporozoite infection. Peak parasitemias in the CD4-depleted vaccinated animals reached nearly 60% (compared to 5% in the non-treated immunized mice) and the time to parasite clearance was extended by more than 3 weeks for the CD4–depleted mice relative to the vaccinated controls. In the same experiment, vaccinated mice were also depleted of CD8 or NK T cells prior to a challenge with PyNL sporozoites. Fig 4B shows mean parasitemia levels for naïve, vaccinated, and cell depleted vaccinated mice at a peak day of parasitemia (day 16 after the infection). In Fig 4B, parasitemia levels were reduced 82% in vaccinated mice compared to naïve controls. However, 2.5 fold (p = 0.04) and 13-fold (p = 0.007) increase in parasitemia percentages relative to naïve and the vaccine groups, respectively, were observed in vaccinated mice depleted of CD4 T cells. In contrast, depletion of CD8 and NK T cells did not impact the levels of protective immunity elicited by immunization with the MVA-CSP/IL15 vaccine.

### Multi-parameter flow cytometry analysis of T cells from vaccinated mice

**Fig 5:**
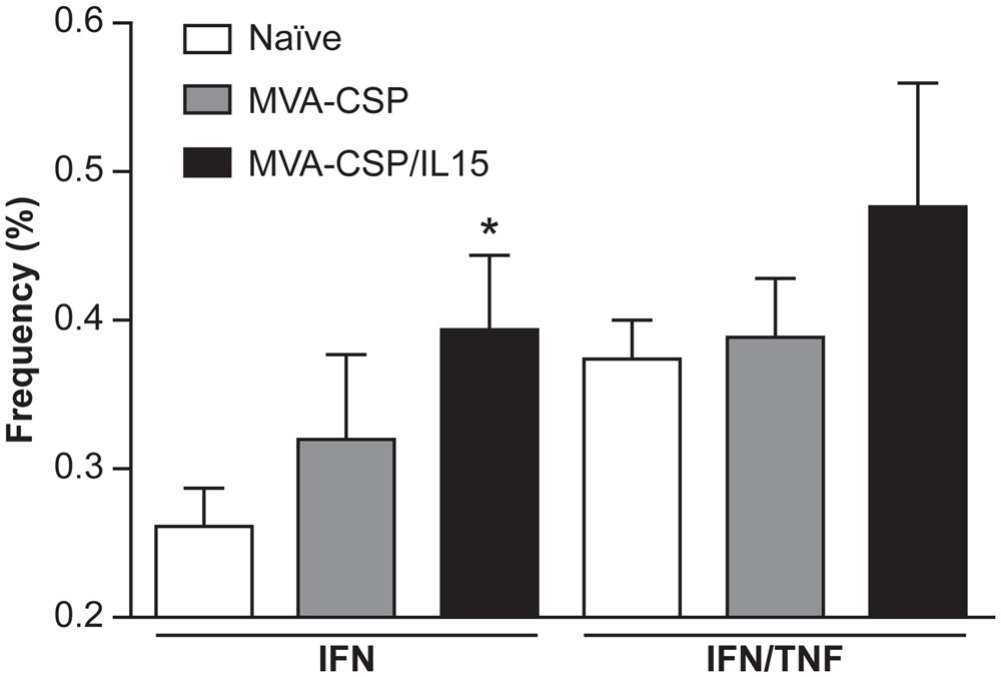
Multi-parameter flow cytometry analysis of splenocytes harvested from vaccinated or sham-vaccinated control mice (4 mice per group). Splenocytes from individual mice immunized with either the MVA-CSP/IL15 vaccine or the MVA-CSP construct and naïve controls were stimulated with recombinant CSP, stained using standard intracellular staining protocols (see Materials and Methods *section* for details) and then analyzed by multi-parameter flow cytometry. The cells were evaluated for the presence of CD3, CD4 and CD8 cell surface markers and the expression of IFN-γ, TNF-α, and/or IL-2. Only the frequencies of cells expressing IFN-γ and IFN-γ/TNF-α are shown. (*p<0.05, relative to naive).

Previous studies have suggested that multifunctional T cells contribute to the vaccine-induced protective immune responses against intracellular pathogens [20, 21]. To evaluate the T cell profile after vaccination with the MVA-CSP or MVA-CSP/IL15 vaccines, spleen cells from vaccinated and naïve mice (10 per group) were harvested one month after the second vaccination, stimulated for 48 hours with recombinant CSP (full-length polypeptide), and evaluated using intracellular cytokine analysis protocols. In particular, we focused on assessing CD4 and CD8 T cell subsets that expressed either IFN-γ, IFN-γ and TNF-α, IFN-γ and IL-2, TNF-α and IL-2, or all three cytokines. As shown in Fig 5, the MVA-CSP/IL15 vaccine induced moderate frequencies of CD4 T cells expressing IFN-γ (p<0.05) and IFN-γ/TNF-α relative to naïve controls. The MVA-CSP vaccine only evoked a modest CD4 T cell IFN-γ response. Furthermore, no significant vaccine-induced T cell responses were seen in any of the CD8 T cell subsets that were analyzed. Overall, no statistical differences in the frequencies of CD4 and CD8 T cells expressing relevant cytokines were detected between the two MVA-based vaccines.

### The MVA-CSP/IL15 vaccine induces elevated anti-CSP humoral responses

As recent studies have demonstrated that protection evoked by the CSP-based RTS,S vaccine in humans correlates with high levels of anti-CSP IgG antibodies, we determined the extent of humoral responses induced by the MVA-CSP/IL15 vaccine (Table 1). As controls, the levels of anti-CSP IgG and IgM antibodies in mice immunized with the MVA-CSP construct and naïve controls were evaluated. For these studies, sera from 10 mice were pooled 2 weeks after the final vaccination and endpoint titers were determined by ELISA using recombinant full-length CSP protein as the coating antigen. Although the total IgM concentrations were similar for both vaccine groups (endpoint titers of 1:400), the levels of total IgG anti-CSP antibody were elevated 100-fold more for the MVA-CSP/IL-15 group (2.0x10^6^ endpoint titer) compared to mice immunized with the MVA-CSP vaccine that did not express IL-15 (endpoint titer-1.3x10^4^). Among the IgG subtypes shown in Table 1, the IL-15 expressing MVA construct induced substantially higher levels of IgG1 (2.6 x10^4^) and IgG2b (5.1x10^5^) than the MVA-CSP vaccine (3.2x10^3^ and 6.4x10^3^, respectively). While the anti-CSP IgG2c antibody concentrations were considerably lower in both groups, increased IgG2c levels (3.2 x 10^3^) were also observed for the MVA-CSP/IL15 vaccine compared to the MVA-CSP construct (2.0 x 10^2^).

**Table 1:** Anti-CSP antibody titers in vaccinated mice.

Antibody Isotype	Anti-CSP Titer	
	MVA-CSP/IL-15^a^	MVA-CSP
**IgG**	**2.0 x 10** ^**6**^	**1.3 x 10** ^**4**^
**IgG1**	**2.6 X 10** ^**4**^	**3.2 x 10** ^**3**^
**IgG2b**	**5.1 x 10** ^**5**^	**6.4 x 10** ^**3**^
**IgG2c**	**3.2 x 10** ^**3**^	**2.0 x 10** ^**2**^
**IgG3**	**4.0 x 10** ^**2**^	**2.0 x 10** ^**2**^
**IgM**	**4.0 x 10** ^**2**^	**4.0 x 10** ^**2**^

^a^ Mice (n = 10) were vaccinated twice, 4 weeks apart and the sera were collected two weeks after the second dose of vaccine and pooled. The anti-CSP titers were defined as the serum dilution (pooled sera from 10 mice in group, single experiment) which generated a mean experimental absorbance value (+ 3 standard deviations) which did not exceed the naïve value for the corresponding dilution.

## Discussion

Recent clinical studies have shown that the most advanced malaria vaccine, RTS,S formulated in AS01 adjuvant, induces 30–50% protection in young African children [6, 7]. While this result represents a seminal achievement for malaria subunit vaccines, it is clearly only an initial step in the development of a highly efficacious immunization strategy against malaria. Since heterologous prime/boost protocols have been shown to evoke potent and persistent cellular and humoral responses against intracellular pathogens, a promising new approach in malaria vaccinology would be to generate strong agents to boost primed RTS,S responses [9]. It should be emphasized that homologous boosting with the RTS,S vaccine did increase vaccine efficacy in 5–17 months old children during a 48-month post-immunization time period but the overall efficacy for this boosting strategy was still only 38%. In addition, only modest RTS,S boosting to a vaccine efficacy of 25.9% was seen in the 6–12 weeks old infants during the duration of the RTS,S phase 3 trial [7]. For heterologous boosting, recombinant MVA constructs are among the most potent vaccines and are capable of inducing highly elevated immune responses in animals and humans [14–16]. Importantly, smallpox vaccination studies in more than 100,000 individuals have shown that these replication-deficient MVA vaccines are safe [20]. However, in a disappointing earlier prime/boost study in 18 adults, boosting a RTS,S/adjuvant formulation with a MVA construct expressing the *P*. *falciparum* CSP elicited enhanced immune responses but not improved protection against an experimental malaria challenge [17].

To potentially improve CSP responses from MVA-based vectors, we generated a recombinant MVA vaccine which co-expresses CSP and IL-15. Previous studies in TB, HIV, and influenza animal models have shown that MVA-based vaccines expressing pathogen-specific antigens as well as IL-15 elicited strong and durable protective immune responses [10, 11, 21]. In this study, we have demonstrated that the IL-15 containing MVA-CSP vaccine induces substantially higher levels of anti-CSP IgG antibody and most importantly, vaccinated mice had a reduced parasite burden through the course of infection and reached significantly lower peak parasitemia and had an accelerated parasite clearance against a sporozoite challenge than a similar MVA-CSP construct which did not express IL-15.

A CSP-based vaccine is thought to target pre-erythrocytic stage parasites by either generating neutralizing antibodies that prevent the invasion of sporozoites into liver cells, or by inducing T cells that recognize and destroy parasites developing inside the liver cells. We find that the MVA-CSP/IL-15 vaccinated mice had a 78% lower liver stage parasite burden than did non-immunized mice suggesting that vaccination-induced immunity was highly effective in reducing the number of liver stage parasites and thereby resulting in fewer liver stage merozoites released into the circulation. We think that the lower merozoite counts, along with the possible immunostimulatory effect of IL-15 may have been responsible for the reduced parasite burden observed in our study. Previously, IL-15 was shown to support the early control and resolution of parasitemia in mice infected with *P*. *chabaudi* AS parasites [22]. In field studies, lower parasite burden is associated with less severe disease and mortality. In fact, RTS,S the most successful sub-unit based malaria vaccine, is also known to reduce severe disease without inducing sterilizing immunity by reducing exposure to blood stage parasites [23]. Interestingly, ownership of insecticide-treated mosquito nets in several Sub-Saharan African countries has been shown to be associated with a reduction of mortality in children 1 month to 5 years of age despite only a modest decrease (23%) in parasitemia [23]. Thus, MVA-based, IL-15 coexpressing candidate malaria vaccines may have an impact on malaria-related morbidity and mortality in endemic areas by reducing levels of blood-stage parasites despite an inability to induce sterile immunity.

Although the enhanced immunogenicity of the MVA-CSP/IL15 vaccine is clear, the mechanisms responsible for IL-15 associated elevated immune responses are uncertain. Since IL-15 is known to have numerous immunomodulatory activities including the activation of B, T and NKT cells and the maintenance of memory cells, the immune enhancing mechanisms may be complex [19]. Recent studies have shown that the ability of IL-15 to enhance dendritic cell maturation contributes to its effective adjuvant-like properties. For instance, Saikh et al concluded that an IL-15 adjuvant increased the effectiveness of a recombinant staphylococcal enterotoxin vaccine by increasing protective humoral responses; IL-15 induced dendritic cell maturation contributed to these enhanced protective antibody levels [24]. IL-15 in combination with CD40 ligand elicited potent polyclonal IgG1 secretion while IL15 and CpG oligonucleotides induced the proliferation of class switched human memory B cells [25, 26]. Furthermore, membrane bound IL-15 has been shown to influence germinal center B cell interactions [25]. Elucidating the specific mechanisms associated with the adjuvant-like properties of IL-15 remains a subject of ongoing investigations.

Cell depletion studies showed that CD4 (but not CD8 and NK) T cells significantly contribute to the MVA-CSP/IL15 vaccine-induced protection. This result is not surprising since CD4 T cells have been shown to play a pivotal role in protecting against blood stage malaria infections. Studies in mice and human have indicated that both the helper and effector functions of CD4 T cells contribute to immunity against malaria [27–29]. Cell-mediated immunity can clearly control parasitemia growth with IFN-γ being involved in the killing of parasites. However, our flow cytometry analysis indicated that only modest IFN-γ T cell responses were induced by vaccination and that MVA-CSP and MVA-CSP/IL15 constructs induced similar cytokine responses. In contrast, the striking 100-fold increased anti-CSP IgG antibody responses detected in mice vaccinated with the MVA-CSP/IL15 vaccine compared to the MVA-CSP construct is likely critical to the enhanced protection seen with the IL-15 containing vaccine. In particular, the elevated levels of IgG1 and IgG 2b (Th-2 type) antibodies detected in mice immunized with the MVA-CSP/IL-15 vaccine may be responsible for the increased protection seen with this IL-15 expressing vaccine. The importance of antibodies in protecting against human malaria was first demonstrated in the passive transfer studies of Cohen et al [30]. For mouse malaria, the passive transfer of hyperimmune serum from mice that had resolved a non-lethal *P*. *yoelii* infection was subsequently shown to protect naïve recipients against a *P*. *yoelii* challenge [31]. Similar to our findings, elevated IgG1 and IgG2b antibody levels were associated with protection in this mouse study. More recently, studies have indicated that the induction of high levels of anti-CSP antibodies correlated with vaccine-induced protection against malaria challenges in mouse models [32]. Interestingly, in a humanized mouse model, passive transfer of human monoclonal antibodies induced by RTS,S vaccination prevented *P*. *falciparum* infection [33]. Overall, anti-CSP antibodies seem to be necessary and possibly sufficient to protect mice against malaria. Recent analysis of RTS,S clinical data has also indicated that anti-CSP antibody responses are surrogates of protection for the magnitude and duration of vaccine efficacy in young children [34, 35]. In humans, a threshold level of anti-CSP specific antibodies seems to be required to evoke protective responses and very high antibody levels are needed to generate sterilizing immunity.

It is intriguing to note that despite the presence of pre-existing anti-CSP antibodies and coupled with the fact that in the MVA-CSP/IL-15 vaccinated mice, the parasitic burden in the liver was markedly reduced, yet we failed to detect any delay in the patency of *P*. *yoelii* or any reduction in the magnitude of parasitemia in the early phase of infection in the MVA-CSP/IL-15 vaccinated mice. One possibility is that we may have missed potential early differences because our first measurement of parasitemia was done 7 days post-challenge or alternatively, the sensitivity/resolution of the Giemsa staining/microscopy we used was not high enough to accurately quantify early subtle differences.

Clearly, immunization strategies are needed to boost the levels and durability of protective humoral and cell-mediated responses induced by candidate malaria vaccines. Disappointingly, in the earlier RTS,S prime-boost study, the heterologous boosting with the MVA-CSP construct did not induce increased antibody responses or enhanced protection against an experimental *P*. *falciparum* malaria challenge [17]. Given the capacity of the MVA-CSP/IL-15 vaccine to evoke more potent humoral responses than the MVA-CSP construct and the importance of generating a threshold anti-CSP response for protection, evaluating a RTS,S prime and MVA-CSP/IL-15 boost vaccination strategy to enhance the effectiveness and persistence of RTS,S-based immunizations is warranted.
